# Macrophage S1PR1 Signaling Alters Angiogenesis and Lymphangiogenesis During Skin Inflammation

**DOI:** 10.3390/cells8080785

**Published:** 2019-07-28

**Authors:** Shahzad Nawaz Syed, Rebecca Raue, Andreas Weigert, Andreas von Knethen, Bernhard Brüne

**Affiliations:** 1 Institute of Biochemistry I, Faculty of Medicine, Goethe-University Frankfurt, 60590 Frankfurt, Germany; 2 Project Group Translational Medicine and Pharmacology TMP, Fraunhofer Institute for Molecular Biology and Applied Ecology, 60596 Frankfurt, Germany; 3 German Cancer Consortium (DKTK), Partner Site Frankfurt, 60590 Frankfurt, Germany; 4 Frankfurt Cancer Institute, Goethe-University Frankfurt, 60596 Frankfurt, Germany

**Keywords:** macrophage, S1PR1, sphingosine-1-phosphate, psoriasis, inflammation, lymphangiogenesis, angiogenesis

## Abstract

The bioactive lipid sphingosine-1-phosphate (S1P), along with its receptors, modulates lymphocyte trafficking and immune responses to regulate skin inflammation. Macrophages are important in the pathogenesis of psoriasiform skin inflammation and express various S1P receptors. How they respond to S1P in skin inflammation remains unknown. We show that myeloid specific S1P receptor 1 (S1PR1) deletion enhances early inflammation in a mouse model of imiquimod-induced psoriasis, without altering the immune cell infiltrate. Mechanistically, myeloid S1PR1 deletion altered the formation of IL-1β, VEGF-A, and VEGF-C, and their receptors’ expression in psoriatic skin, which subsequently lead to reciprocal regulation of neoangiogenesis and neolymphangiogenesis. Experimental findings were corroborated in human clinical datasets and in knockout macrophages in vitro. Increased blood vessel but reduced lymph vessel density may explain the exacerbated inflammatory phenotype in conditional knockout mice. These findings assign a novel role to macrophage S1PR1 and provide a rationale for therapeutically targeting local S1P during skin inflammation.

## 1. Introduction

Psoriasis is a common inflammatory disease of the skin, with clear genetic determinants, affecting around 1.5–3% of the European population [1]. Inflammatory stimuli in genetically susceptible individuals are the predominant trigger to initiate or exacerbate psoriatic lesions [2]. Many psoriatic mouse models require autoreactive T cells and dendritic cells (DC) for the development of skin inflammation [3]. However, there is ample evidence in the literature that the intrinsic defect in psoriasis may lie well within the psoriatic skin, with the overproduction of inflammatory mediators such as TNFα triggering and perpetuating inflammatory reactions, independent of autoreactive T cells [4,5]. Elegant studies, in either T cell-[5] or skin-dependent [4] models of psoriasis, established the critical role of macrophages as a source of TNFα, substantiated by anti-TNFα rescue studies or depletion of macrophages. Recently, it has been demonstrated in a mouse line with inducible transgenic overexpression of human TNF, that macrophages, even in the absence of T and B cells, are sufficient for inducing psoriasis and are counter-regulated by Treg [6]. Macrophages are multitasking and dynamic cells that respond to various environmental cues and adapt accordingly [7]. Thus, it appears rational that macrophages may contribute to skin inflammation in a much more complex way than being merely a source of TNFα.

In an inflammatory microenvironment, macrophages encounter various factors derived from apoptotic cells and activated neighboring cells. One such factor is sphingosine-1-phosphate (S1P), which is released by apoptotic cells and cells in the inflamed skin [8,9]. S1P is a bioactive lipid that regulates vascular morphogenesis, endothelial permeability, leukocyte adhesion, and inflammation [9,10]. Macrophages express various S1P receptors (S1PR) and are involved in pathophysiological processes integral to inflammation [11,12]. Research on the role of S1PRs in inflammatory diseases, such as psoriasis, is also gaining attention. Notably, a phase 2 trial showed a beneficial effect of the oral S1P analog ponesimod in patients with chronic plaque psoriasis. This was attributed to S1PR1 internalization on inflammatory T and B cells, which rendered these cells insensitive to the concentration gradient of S1P and thus, prevented egress of lymphocytes from secondary lymphoid tissues [13]. In an imiquimod (IMQ), a toll-like receptor 7 (TLR7) agonist, induced mouse model of psoriasis, S1P exhibited anti-proliferative effects on dermal cells and anti-inflammatory effects by preventing immune cell, particularly DC, infiltration [14]. Nevertheless, our understanding of the impact of macrophage S1PR signaling in skin inflammation is very limited. Recently published literature and clinical studies highlighted the critical involvement of macrophages and S1P in inflammation-associated lymphangiogenesis (IAL). However, there was no consensus on the benefit of S1P in IAL, partly due to a lack of understanding of the molecular mechanisms or due to the diverse pathophysiological findings attributed to S1P. Accumulating evidence suggests that IAL is not merely an endpoint of inflammation, but rather a dynamic, context-dependent process that can alter the progression of inflammation and/or tissue repair. In this context, the role of IL-1β in lymphangiogenesis is very well established [12,15]. However, it is unclear how and in which cell type the inflammasome is activated to release IL-1β, especially in the settings of psoriatic IAL. Intriguingly, increased permeability of lymphatic capillaries, increased blood flow, and angioproliferation are characteristic features of psoriatic lesions [16]. Based on our previous findings indicating that S1PR-dependent IL-1β production promotes lymphangiogenesis in tumors [12], we hypothesized that macrophage S1PR1 signaling might promote neolymphangiogenesis and tissue repair. Hence, macrophage S1PR1 signaling might be beneficial in psoriasis.

In the present study, we reached out to test our hypothesis in a mouse model of myeloid-specific S1PR1 deletion by imiquimod (IMQ)-induced psoriasiform inflammation in conjunction with human clinical datasets.

## 2. Materials and Methods

### 2.1. Mice and Reagents

*S1pr1^loxP/loxP^* (B6.129S6(FVB)-*S1pr1^tm2.1Rlp^*/J) mice have been described before [17] and were crossed with *Hif1a^fl/fl^-Lyz2^Cre/Cre^* mice [18]. Littermates were backcrossed for several generations to obtain *Hif1a^wt/wt^S1pr1^fl/fl^Lyz2^Cre/Cre^* (*S1pr1^∆MΦ^*) or *Hif1a^wt/wt^S1pr1^fl/fl^Lyz2^Cre/wt^* (*S1pr1^wtMΦ^*) on a C57BL/6 background. For all mice the genotypes were determined by PCR analysis of tail genomic DNA. Mice were kept under standard pathogen-free conditions with food (regular chow diet) and water ad libitum and a 12:12 h light–dark cycle. Ultrapure lipopolysaccharide (LPS) from *E*. *coli* 0111:B4 was purchased from InvivoGen (San Diego, CA, USA) and dimethyl sulfoxide (DMSO) was purchased from Sigma-Aldrich (München, Germany). The source of all other reagents is mentioned at their respective use.

### 2.2. Analysis of Publicly Available Datasets of Gene Expression in Psoriasis Patients

Gene Expression Omnibus (GEO) datasets comprising 85 patients (GSE30999) and 58 patients (GSE13355) were downloaded and analyzed either via GEO2R (https://www.ncbi.nlm.nih.gov/geo/geo2r/) or GDSBrowser (https://www.ncbi.nlm.nih.gov/sites/GDSbrowser/). Expression profiles of S1P metabolizing enzymes and receptors, along with angiogenesis and lymphangiogenesis markers in normal adjacent and psoriatic tissues from patients, were further analyzed and plotted using GraphPad Prism v8 (GraphPad Software, San Diego, CA, USA). 

### 2.3. Psoriasiform Dermatitis Model

All animal experiments were conducted with approval from the local ethical review committee and in accordance with the guidelines of the Hessian animal care and use committee (approval no. FU/1154). For imiquimod (IMQ)-induced psoriasis experiments, mice were anaesthetized with isoflurane. Mouse back skin was shaved one day before starting the experiment. 62.5 mg of commercially available cream containing 5% IMQ (Aldara; 3M Pharmaceuticals) or vaseline (control) were daily applied on the back skin and inner right ear of 8–12 weeks old male or female mice for 2, 3, or 5 consecutive days. The severity of the skin inflammatory response was assessed on the basis of the Psoriasis Area Severity Index (PASI) as described previously [19,20]. Briefly, the three parameters of psoriasis responses—erythema, scaling, and skin thickness—were scored independently on a scale from 0 to 4 as follows: 0: none; 1: slight; 2: moderate; 3: marked; and 4: very marked. By adding up the scores from these three parameters, the severity of the response was measured using a cumulative score from 0 to 12. Mice were sacrificed, and skin as well as plasma samples were harvested on day 2, 3 or 5 for further analyses.

### 2.4. RNA Isolation, Reverse Transcription, and Quantitative Real-Time PCR

Mouse skin was snap frozen in liquid nitrogen and homogenized either directly in PeqGold^®^ (Peqlab Biotechnology, Erlangen, Germany) using the Precellys^®^ 24 tissue homogenizer (Bertin Instruments, Montigny-le-Bretonneux, France) or by ceramic pestle and mortar before resuspension in PeqGold. Isolation of RNA from skin tissue and cells was performed according to the manufacturer’s instructions and quantified using the NanoDrop spectrophotometer (NanoDrop, Wilmington, USA). RNA was transcribed into cDNA for mRNA analysis using the Fermentas reverse transcriptase kit (ThermoFisher Scientific, Karlsruhe, Germany) according to the manufacturer’s instructions. Real-time quantitative PCR (qPCR) was performed using the SYBR green on CFX96™ Real-Time PCR Detection System (Bio-Rad Laboratories, Munich, Germany) and the QuantStudio 5 Real-Time PCR System (Applied Biosystems, Damstadt, Germany). QuantiTect primer assays (QIAGEN, Hilden, Germany) were used to detect murine *Kdr*, *Vegfa, Vegfc, Mrc1, S1pr2, S1pr3*, and *S1pr4*. All other primers were from Biomers GmbH, Germany, and their sequences are presented in Table S1. Relative mRNA expression was calculated using either the CFX-Manager^TM^ v3.2 software (Bio-Rad Laboratories) or the QuantStudio^TM^ Design and Analysis software v1.5 (Applied Biosystems) using the ^ΔΔ^Ct method and normalized to either *Actb* or *Rps27a* as housekeeping genes. 

### 2.5. Flow Cytometry

Skin tissue was chopped into small pieces and incubated in 300 μg/mL liberase and 50 U/mL DNase l (Merck, Damstadt, Germany) for 90 min to obtain single cell suspensions. Single cell suspensions were blocked with FcR blocking reagent (Miltenyi Biotec, Gladbach, Germany) in 0.5% PBS-BSA for 20 min and stained with fluorochrome-conjugated antibodies. For characterization of immune cell subsets in skin the following antibodies were used: anti-CD3-PE-CF594, anti-CD4-BV711, anti-CD8-BV650, anti-CD11b-BV605, anti-CD11c-AlexaFluor700, anti-CD19-APC-H7, anti-CD326-BV711, anti-Ly6C-Per-CP-Cy5.5, anti-NK1.1 BV510 (BD Biosciences, Heidelberg, Germany), anti-CD45-Vio-Blu, anti-MHC-II-APC (Miltenyi Biotec), anti-CD-90.2-PE, anti-F4/80-PE-Cy7, anti-GITR-FITC, anti-γδ TCR APC, and anti-Ly6G-APC-Cy7 (BioLegend, San Diego, CA, USA). For characterization of endothelial cells, single cell suspensions were further stained with anti-CD31-PE-Cy7, anti-CD44-AlexaFluor700, anti-CD49f-PE-CF594 (BD Biosciences), anti-CD117-APC-eFluor780 (ThermoFisher) anti-CD140-PE, anti-CD146-AlexaFluor488, and anti-CD324-AlexaFluor647 (BioLegend). Stained cells were analyzed on a LSR II/Fortessa flow cytometer (BD Biosciences). Data were analyzed using FlowJo Vx (Tree Star, Inc., Ashland, OR, USA). All antibodies and secondary reagents were titrated to determine optimal concentrations. Comp-Beads (BD Biosciences) were used for single-color compensation to create multicolor compensation matrices. For gating, fluorescence minus one controls were used. The instrument calibration was controlled daily using Cytometer Setup and Tracking beads (BD Biosciences).

### 2.6. Multiplex Immunohistochemistry

Formaldehyde fixed, paraffin embedded sections were sequentially stained with antibodies against mouse CD163 (Abcam, #ab182422; 1:250 dilution), CD31 (Cell Signaling, #77699; 1:500 dilution), and LYVE-1 (R&D Systems, #AF2125; 1:200 dilution) using the Opal staining system according to the manufacturer’s instructions (Perkin Elmer). Nuclei were counterstained with DAPI and slides were mounted with Fluoromount-G (SouthernBiotech, Birmingham, USA). The Vectra^®^ 3 automated quantitative pathology imaging system (PerkinElmer, Rodgau, Germany) was used for image acquisition at 20× and images were analyzed using the inForm2.0 Software (PerkinElmer) and ImageJ as follows. Composite images of whole tissue sections were RGB stacked. The threshold of the DAPI signal was set for positive identification of all cells in the frame. Set threshold values were used for quantification of pixels in each channel. Data are presented as percentage area occupied by stain of interest in all images of a tissue by DAPI stain.

### 2.7. Cytometric Bead Array

To determine cytokine levels in cell culture supernatants or murine skin (as described before [12]), murine IL-17A, IL-1β, IL-6, IL-23, CCL5, and CCL2 Cytometric Bead Array (CBA) Flex Sets (BD Biosciences) were used. Mouse skin tissue supernatant was prepared by homogenization of snap frozen tissue using ceramic pestle and mortar and extracted in modified RIPA buffer (50 mM Tris/HCl pH 7.5, 1% Triton X-100, 0.5% sodium deoxycholate, 0.1% sodium dodecyl sulphate, 150 mM NaCl, complete EDTA-free protease inhibitor cocktail (Roche, Mannheim, Germany), and 1 mM PMSF). Samples were acquired with an LSR Fortessa flow cytometer (BD Biosciences) and data was analyzed using the BD Biosciences FCAP software (V3.0).

### 2.8. Macrophage Culturing and Stimulation

Bone marrow was isolated from the tibia and femur of *S1pr1^∆MΦ^* or *S1pr1^wtMΦ^* mice and 4 × 10^6^ bone marrow cells per well of a 6-well plate were incubated in RPMI 1640 containing 10% FCS, 100 U/mL penicillin, 100 µg/mL streptomycin, 20 ng/mL M-CSF and 20 ng/mL GM-CSF (ImmunoTools, Friesoythe, Germany) for 7 days. Medium was replaced every 2 days. Peritoneal macrophages (PM) were isolated from the peritoneal cavity of naïve mice by flushing it with 5 mL ice-cold PBS. 1 × 10^6^ peritoneal cells were cultured in wells of a 48 well plate. After 3 h, non-adherent cells were washed away and adherent PMs and differentiated bone marrow-derived macrophages were stimulated with 100 ng/mL of LPS and 10 μg/mL IMQ for 6 h.

### 2.9. Genotyping PCR

Genomic DNA from *S1pr1^∆MΦ^* or *S1pr1^wtMΦ^* bone marrow-derived macrophages (BMDMs) was isolated using Proteinase K (20 mg/mL) containing lysis buffer (10 mM EDTA, 10 mM NaOH, 100 mM Tris, pH 9) for 20 min at 50 °C and neutralized in 100 mM Tris buffer pH 4 containing 1.5% BSA. A KAPA mouse genotyping kit (Kapa Biosystems, Wilmington, USA) was used for PCR according to the manufacturer’s instruction. Published primers for *S1pr1* loxP [17] and *Lyz2*-Cre [21] were used for PCR with 5 min denaturation at 95 °C with 35 cycles of 20 s denaturation at 95 °C, 15 s annealing at 60 °C, and 20 s extension at 72 °C. Bands were resolved on a 2% agarose gels and photographed using the Smart3 Gel documentation system (VMR, Darmstadt, Germany). 

### 2.10. Statistical Analysis 

All data are presented as mean values ± SD of at least two independent experiments. Statistical analyses were performed in GraphPad Prism v8 using two-tailed Student’s *t*-test, two-way ANOVA with Bonferroni’s correction, and Pearson correlation coefficients with two-tailed *p* values, as indicated in the figure legends. Asterisks indicate significant differences between experimental groups (* *p* < 0.05, ** *p* < 0.01).

## 3. Results

### 3.1. S1PR1 is Downregulated in Human Psoriatic Patients

**Figure 1:**
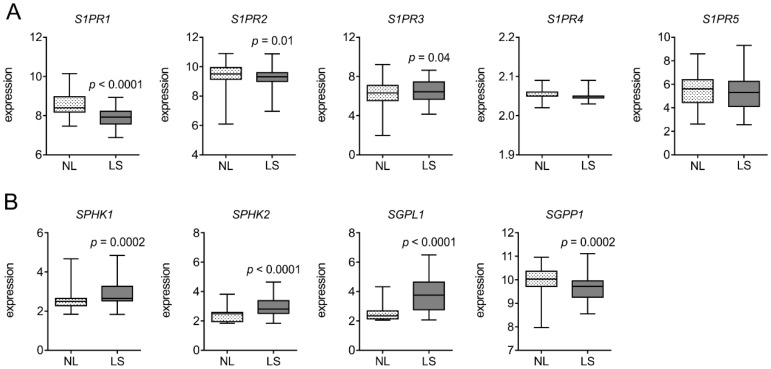
Sphingosine-1-phosphate receptor 1 (*S1PR1*) is downregulated in human psoriatic patients. Gene expression data in Gene Expression Omnibus (GEO) dataset GSE30999 (21) were analyzed for (**A**) expression of S1P receptors and (**B**) S1P metabolizing enzymes in tissues from psoriatic patients with (LS) and without (NL) lesions (*n* = 85). *p* values were calculated using two-tailed Student’s *t*-test.

To understand the relevance of S1PR1 in psoriasiform skin inflammation, we first scanned its expression pattern in GEO datasets. A microarray dataset containing gene expression profiling of skin biopsy samples from 85 patients (GSE30999) with moderate-to-severe psoriasis [22] was used (Figure 1). Another microarray dataset, deposited by the Collaborative Association Study of Psoriasis (CSAP) [23], with data on gene expression in psoriasis lesions compared with matched biopsies of non-lesional skin from 58 patients (GSE13355) was also analyzed for S1PR expression (Figure S1). Among the five known S1P receptors, only *S1PR1* was downregulated in psoriatic lesion compared to matched non-lesional skin (*n* = 85, *p* < 0.0001; Figure 1A and *n* = 58, *p* = 0.0004; Figure S1A). The expression of S1P metabolizing enzymes was also altered (Figure 1B and Figure S1B). Kinases required for S1P production (*SPHK1* and *SPHK2*) were upregulated (*p* < 0.001) along with S1P lyase 1 (*SGPL1*), whereas S1P phosphatase 1 (*SGPP1*) was downregulated (*p* = 0.0002). These datasets indicated a correlation between *S1PR1* expression and psoriasis and highlighted the downregulation of S1PR1 during this disease.

### 3.2. Myeloid-Specific S1PR1 Deletion Enhanced Early Inflammation in IMQ-Induced Psoriasis

**Figure 2:**
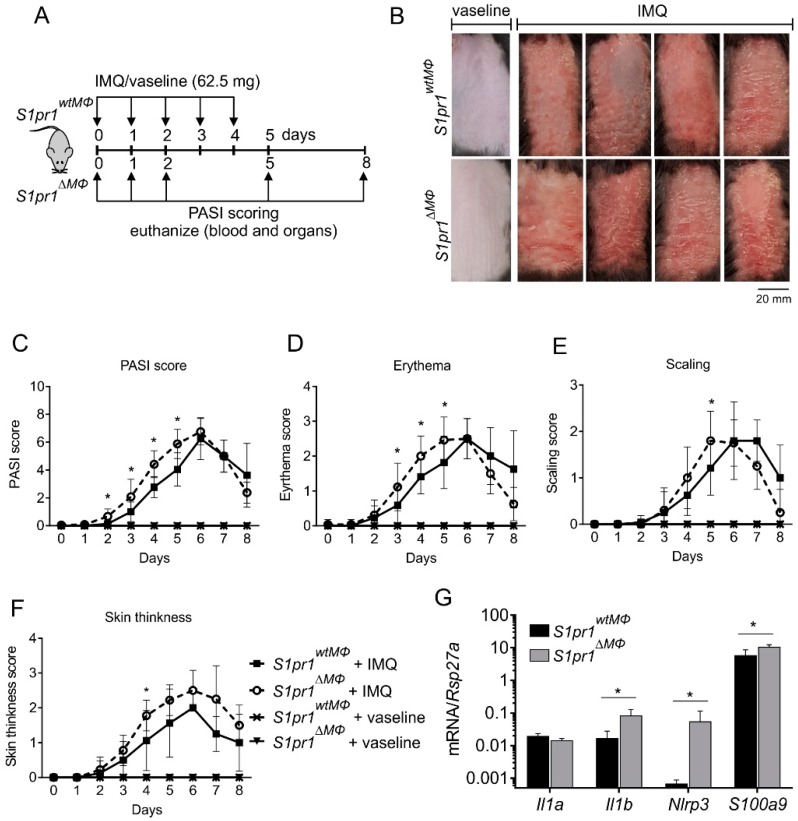
Myeloid *S1pr1* deletion enhanced inflammation in psoriasiform skin inflammation*. S1pr1^wtMΦ^* and *S1pr1^∆MΦ^* mice were treated with either vaseline or 62.5 mg Aldera cream containing 5% imiquimod (IMQ) every day for 5 consecutive days and monitored for another 3 days. (**A**) Schematic representation of IMQ-induced psoriasis model. (**B**) Back skin pictures at day 5 post treatment. (**C**–**F**) Cumulative Psoriasis Area Severity Index (PASI) scores were calculated (**C**) based on the individual scores for erythema (**D**), scaling (**E**), and thickness (**F**) daily until 8 days after initial IMQ-application. Data B–F are means ± SD of 11 individual animals each from three individual experiments. (**G**) mRNA expression of IL-1α, IL-1β, NLRP3 and S100A9 is indicated in *S1pr1^wtMΦ^* (black bar) and *S1pr1^∆MΦ^* (grey bar) mice at day 2 by qPCR. Data are means ± SD, *n* = 4 individual animals. * *p* < 0.05; *p* values were calculated using two-tailed multiple *t*-test with Holm–Šídák correction (C–G).

In light of the human clinical data on *S1PR1* and psoriasis, and the fact that macrophages can contribute to both epithelial-based and T cell-mediated pathways of skin inflammation [4,5], we hypothesized that macrophage specific S1PR1 might play an important role in skin inflammation. To test this hypothesis, myeloid-specific [21] *S1pr1* knockout (hereafter referred to as *S1pr1^∆MΦ^*) mice were generated. Similar mice have been described before [24]. S1PR1 deletion was confirmed in bone marrow-derived and peritoneal macrophages (PMs) from *S1pr1^fl/fl^-Lyz2^Cre/wt^* (hereafter referred to as *S1pr1^wtMΦ^*) and *S1pr1^∆MΦ^* (*S1pr1^fl/fl^-Lyz2^Cre/Cre^*) mice (Figure S2A,B). Since S1PRs might be counter regulated on immune cells [25], we analyzed the expression of *S1pr1*–*4* in PMs from *S1pr1^wtMΦ^* and *S1pr1^∆MΦ^* mice upon stimulation with toll-like receptor agonists such as LPS or imiquimod (IMQ). Myeloid-specific *S1pr1* deletion did not provoked counter regulation of *S1pr2-4* in PMs (Figure S2B). Next, psoriasis-like skin inflammation was induced in these mice by applying the IMQ along with vaseline control treatments (Figure 2A). In line with our previous study on the role of macrophage S1PR1 and inflammation-driven lymphangiogenesis in cancer models [12], we expected alterations in the onset and resolution phase of inflammation in this mouse model of IMQ-induced psoriasiform skin inflammation. Unexpectedly, there was aggravated early manifestation of the disease in *S1pr1^∆MΦ^* mice (Figure 2B). This was characterized by significant differences in the cumulative PASI score from day 2 to 5 (Figure 2C), the erythema score from day 3 to 5 (Figure 2D), scaling at day 5 (Figure 2E), and skin thickness at day 4 (Figure 2F) compared to *S1pr1^wtMΦ^*. However, there was no significant difference in terms of resolution of inflammation and both strains demonstrated overlapping kinetics of inflammation resolution from day 6 onwards as reflected by the PASI score (Figure 2C). Next, expression of *S1pr1*–*4* was analyzed in psoriatic skin by qPCR to assess a potential counter-regulation. In line with human clinical data, *S1pr1* was downregulated in psoriatic skin, whereas *S1pr2* and *S1pr4* remained unaffected. Other potential compensatory mechanisms mediated by S1PRs cannot be ruled out in skin inflammation since *S1pr3* was downregulated in *S1pr1^∆MΦ^* mice compared to *S1pr1^wtMΦ^* in psoriatic mice (Figure S3). There was no inflammation in vaseline-treated control mice of either strain (Figure 2B–F). Therefore, control mice were not analyzed for other parameters, adhering to requirements of the 3R Principle of the German Animal Protection Act. Although PASI scoring was performed in a blind manner, personal bias cannot be ruled out. Hence, to validate the subjective PASI scoring, the expression of inflammation markers such as *S100a9* was measured by qPCR of day 2 psoriatic back skin. This confirmed the enhanced inflammation in *S1pr1^∆M^*^Φ^ mice compared to *S1pr1^wtMΦ^* (Figure 2G). IMQ-induced skin inflammation has been shown to demand IL-1R1 signaling [26]. Therefore, we measured the expression of *Il1b* and *Nlrp3*. Unlike in psoriatic ear skin (Figure S4), these markers were significantly upregulated in back skin, whereas expression of *Il1a* remained unaltered in *S1pr1^∆MΦ^* (Figure 2G). These results pointed to an anti-inflammatory role of myeloid S1PR1 in early psoriasiform skin inflammation in mice, correlating well with human clinical data.

### 3.3. S1pr1^∆MΦ^ Mice Show Reduced Lymph and Increased Blood Vessels

**Figure 3:**
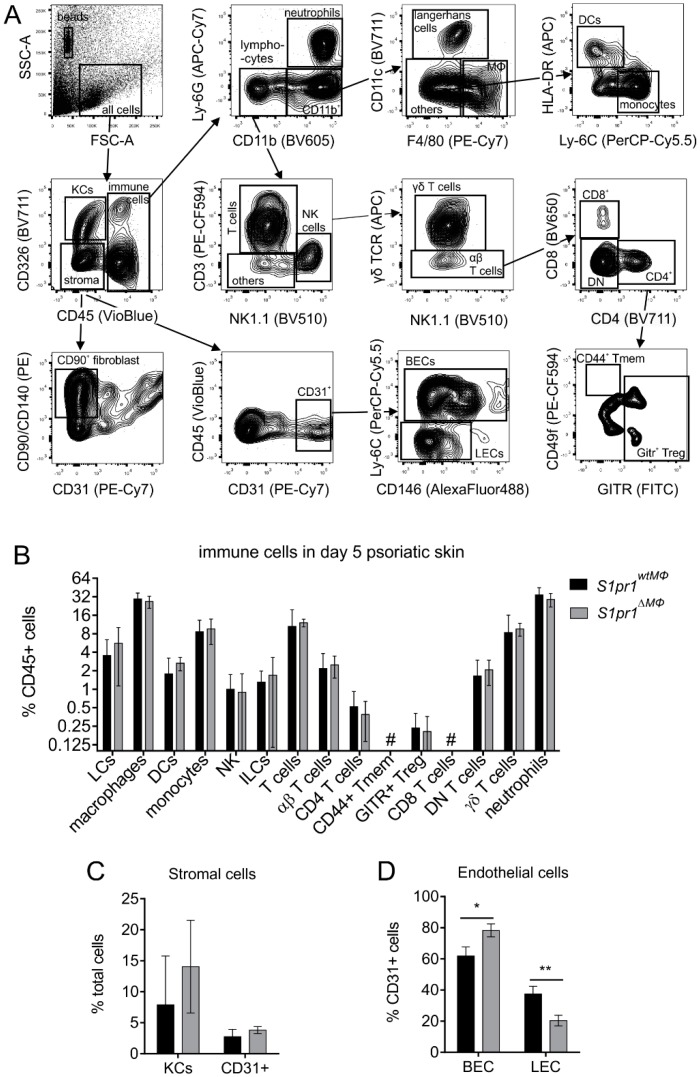
Psoriatic skin cell profile of *S1pr1^wtMΦ^* and *S1pr1^∆MΦ^* mice. Immune and endothelial cell subsets in psoriatic skin of *S1pr1^wtMΦ^* (black bar) and *S1pr1^∆MΦ^* (grey bar) mice at day 5. (**A**) Gating strategy. (**B**) Psoriatic back skin immune cells were separated based on their surface markers. (**C**) Keratinocytes were separated as CD45^-^CD326^+^. Blood and lymphatic endothelial cells were identified in CD45^-^CD326^-^ fraction, which were further separated by (**D**) CD31^+^CD146^+^Ly-6C^+/-^. Data are means ± SD, *n* = 4 individual animals. * *p* < 0.05; ** *p* < 0.001; *p* values were calculated using two-tailed multiple *t*-test with Holm–Šídák correction (C-G). #, below % detection.

Next, we sought to determine the molecular mechanisms underlying enhanced inflammation in *S1pr1^∆MΦ^* mice in the model of IMQ-induced psoriasis. We analyzed total immune cell composition of inflamed skin at day five by flow cytometry with the assumption that immune cell composition might shed some light on cause and effect relations. A panel of 25 markers (Table S2) was used to identify 19 distinct cell types (Figure 3A) in the inflamed back skin. Apart from the tendency of increased dendritic cells (DCs) and monocytes in *S1pr1^∆MΦ^* skin, there were no significant differences in any of the immune cell populations between both strains (Figure 3B) and cellular distribution was also comparable in both strains. Hematopoietic stem cells and plasmacytoid DCs were absent, and CD8^+^ T cells were less than 0.1% of total CD45^+^ cells. Total keratinocytes were slightly increased in *S1pr1^∆MΦ^* skin, which is expected due to hyperproliferation of these cells in response to the inflammation [27], whereas total CD31^+^ stroma cells were unchanged (Figure 3C). Interestingly however, there was a reciprocal regulation of lymphatic endothelial cells (LECs; CD326^+^CD45^-^CD90^hi^CD140^hi^CD31^+^Ly-6C^-^) and blood endothelial cells (BECs; CD326^+^CD45^-^CD90^lo^CD140^lo^CD31^+^Ly-6C^+^) within the CD31^+^ population (Figure 3D). *S1pr1^∆MΦ^* skin had significantly more BECs (*p* = 0.01), but significantly less LECs (*p* = 0.008) compared to inflamed *S1pr1^wtMΦ^* skin. 

**Figure 4:**
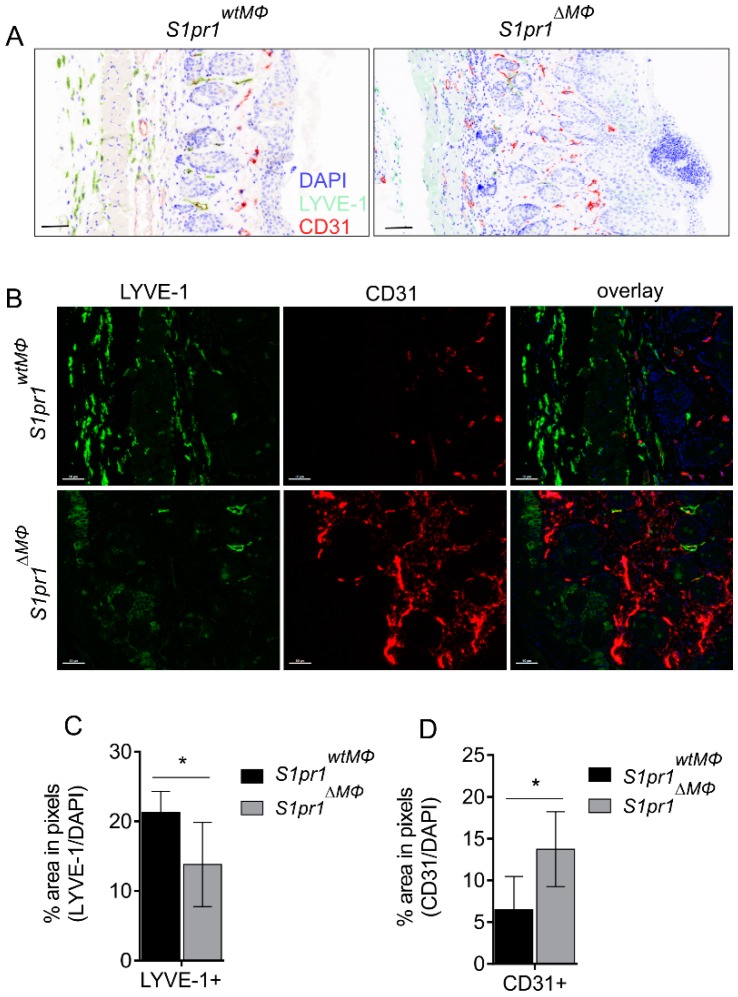
Reciprocal regulation of blood and lymph vessels in psoriatic *S1pr1^∆MΦ^*. (**A**–**D**) *S1pr1^wtMΦ^* (black bar) and *S1pr1^∆MΦ^* (grey bar) mice were treated daily with 62.5 mg IMQ on the back skin for up to 5 days. (**A**) Histology images of skin sections from 5-day IMQ-treated mice (indicative of 7–9 animals each) stained with anti-LYVE-1 (green), anti-CD31 (red) and DAPI (blue). Scale bars represent 100 μm. (**B**) PhenOptics images of skin sections from 5-day IMQ-treated mice (indicative of 7–9 animals each) stained with anti-LYVE-1 (green), anti-CD31 (red) and DAPI (blue). Scale bars represent 50 μm. (**C**) The graph shows quantification of the mean LYVE-1 signal in the whole skin presented as % area in green pixels normalized to blue pixels of nuclear counterstain DAPI. Data are means ± SD, *n* = 6 individual animals. (**D**) The graph shows quantification of the mean CD31 signal in whole skin presented as % area in red pixels normalized to blue pixels of nuclear counterstain DAPI. Data are means ± SD, *n* = 4 individual animals. * *p* < 0.05; *p* values were calculated using two-tailed multiple *t*-test with Holm–Šídák correction (C–D).

To confirm regulation of blood and lymph vessels and to identify morphologically functional vessels, we used a multispectral imaging system [28] (PhenOptics, Perkin Elmer). This method allows automated slide processing and analysis of paraffin fixed sections of day five psoriatic back skin of *S1pr1^wtMΦ^* and *S1pr1^∆MΦ^* mice via tyramide signal amplification for the expression of LYVE-1 (lymphatic endothelial cell marker) and CD31 (blood endothelial cell marker). Immunofluorescence staining of back skin revealed an enhanced acanthosis and enlarged parakeratosis area in *S1pr1^∆MΦ^* compared to *S1pr1^wtMΦ^*, confirming increased inflammation in these animals (Figure 4A). Histomorphology of ear skin was comparable between both strains (Figure S5A), including ear thickness (Figure S5B). In line with data from flow cytometry, there was reduced staining of LYVE-1^+^ LECs in *S1pr1^∆MΦ^* compared to *S1pr1^wtMΦ^* (Figure 4B,C), whereas CD31^+^ BECs were significantly increased in *S1pr1^∆MΦ^* mice back skin (Figure 4B,D). However, there was no difference in blood or lymph vessel density in the ear skin of both of these strains (Figure S5C,D). These results established a relationship between myeloid S1PR1 and reciprocal neoangiogenesis and neolymphangiogenesis.

### 3.4. Human Psoriatic Clinical Data Corroborated a Reciprocal Regulation of Lymph vs. Blood Vessel Markers

**Figure 5:**
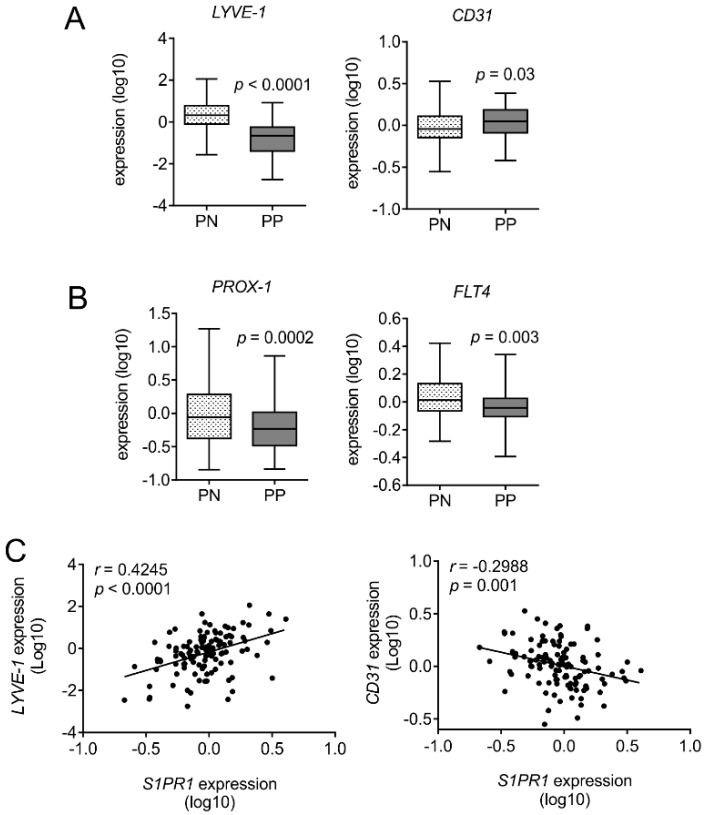
Reciprocal regulation of angiogenesis and lymphangiogenesis in human psoriatic patients. Gene expression data in Gene Expression Omnibus (GEO) dataset GSE13355 [23] were analyzed for (**A**) the expression of the lymphatic marker *LYVE-1* and the blood endothelial cell marker *CD31* as well as (**B**) the expression of the key lymphatic transcription factor *PROX-1* and *FLT4* (encoding VEGFR-3) in tissue with (PP) and without (PN) lesions from psoriatic patients. (**C**) The expression of *LYVE-1*, *CD31,* and *S1PR1* was analyzed to correlate the expression of *S1PR1* with the lymphatic marker *LYVE-1* and the blood endothelial cell marker *CD31*. *p* values were calculated using two-tailed Student’s *t*-test, *n* = 58.

The striking observation of reciprocal regulation of lymph and blood vessels in psoriatic *S1pr1^∆MΦ^* mice encouraged us to explore its clinical relevance in human patient material. We analyzed the expression of lymphatic and blood endothelial markers in adjacent control skin vs. psoriatic skin of 58 patients. Interestingly, substantiating our observations in mice, there was a reciprocal regulation of *LYVE-1* and *CD31* expression (Figure 5A). *LYVE-1* expression was downregulated in psoriatic lesions (PP) in comparison with non-lesions (PN) (*p* < 0.0001), whereas *CD31* expression was enhanced (*p* = 0.03). Furthermore, the key lymphatic transcription factor *PROX-1* and the lymphatic specific receptor *FLT4* (also known as VEGFR-3) were significantly downregulated in psoriatic lesions compared to non-lesions (Figure 5B). Since macrophages in cutaneous squamous cell carcinoma can also express *LYVE-1* [29]. To negate the influence of altered macrophage numbers in psoriatic skin on downregulation of *LYVE-1*, we analyzed its correlation with *S1PR1* expression. The expression of *LYVE-1* was positively correlated with *S1PR1* in non-lesion and psoriatic lesion (Pearson *r* = 0.4245; *p* < 0.0001), whereas *CD31* expression was expectedly in negative correlation with *S1PR1* (Pearson *r* = −0.2988; *p* = 0.03) (Figure 5C). These data consolidated findings of our mouse model of myeloid specific *S1PR1* deletion, further arguing that S1PR1 plays a non-redundant role in inflammation-driven neoangiogenesis and neolymphangiogenesis in the skin.

### 3.5. Reciprocal Angiogenesis and Lymphangiogenesis in S1pr1^∆MΦ^ Is Reflected by Altered VEGFs and Their Receptors

**Figure 6:**
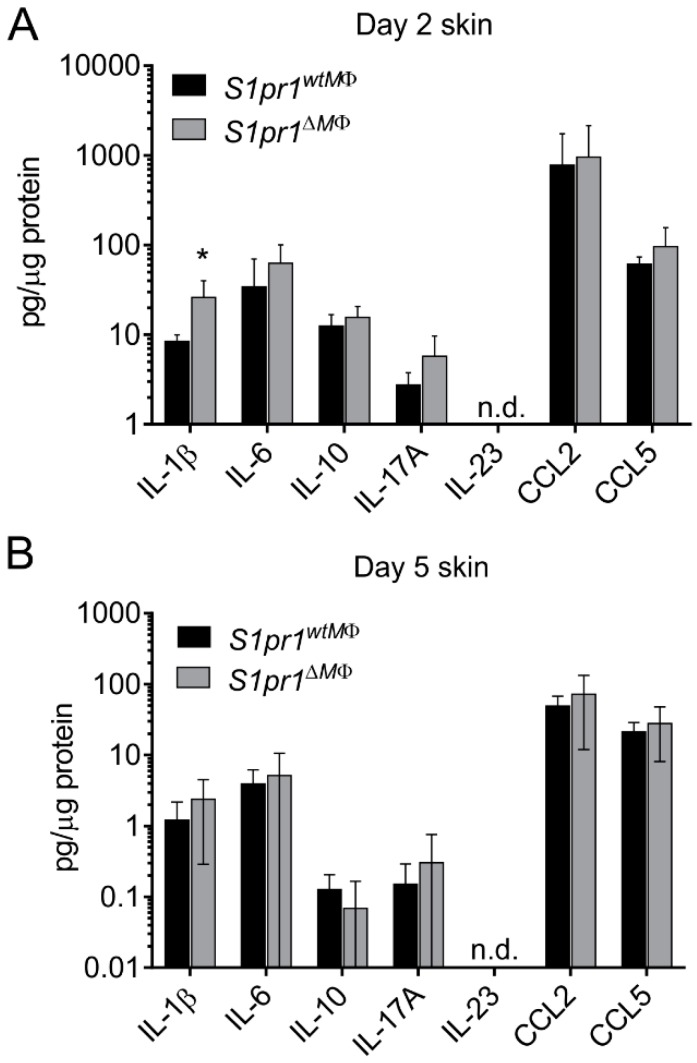
Chemokine and cytokine released in psoriatic skin of *S1pr1^∆MΦ^* mice. (**A**,**B**) *S1pr1^wtMΦ^* (black bar) and *S1pr1^∆MΦ^* (grey bar) mice were treated daily with 62.5 mg IMQ on the back skin for up to 5 days. (**A**) CBA analysis of day 2 IMQ-treated back skin, normalized to protein content of the skin lysate. Data are means ± SD, *n* = 4 individual animals (**B**) CBA analysis of day 5 IMQ-treated back skin, normalized to protein content of the skin lysate. Data are means ± SD, *n* = 7 (*S1pr1^wtMΦ^*) and *n* = 9 (*S1pr1^∆MΦ^*) individual animals. * *p* < 0.05; *p* values were calculated using two-tailed multiple *t*-test with Holm–Šídák correction (C-D). n.d., not detected.

Several factors contribute to angiogenesis and lymphangiogenesis, including pro-inflammatory cytokines, VEGFs, and growth factors. Given that IL-1β directly induces angiogenesis and lymphangiogenesis via VEGF-C induction [12], we next investigated if IL-β or other cytokines were altered in inflamed skin of our mice. Since we observed enhanced inflammation at day two post IMQ-treatment, we selected this time point to analyze cytokine levels in IMQ-treated back skin. Amongst all tested chemokines and cytokines, only IL-1β levels were significantly enhanced in *S1pr1^∆MΦ^* mice compared to *S1pr1^wtMΦ^* mice (26.05 ± 7.02 vs. 8.46 ± 0.73 pg/µg skin supernatant, respectively) (Figure 6A). Enhanced levels of IL-1β at day two are indicative of increased inflammation in *S1pr1^∆MΦ^* mice. Unaltered levels of other cytokines and chemokines at day two inflamed skin supported the observation of Figure 3B, where no alterations in immune cell infiltration were observed at day five inflamed skin. At later time points, i.e., day five post treatment, the level of all cytokines and chemokines dramatically decreased, suggesting a resolution phase of inflammation (Figure 6B). Interestingly, we did not observe differences in IL-17A at day two or day five in these mice, suggesting that the Th17 cell-mediated etiology of this model [19,30] remained unaltered and may not be involved in the phenotype of *S1pr1^∆MΦ^* mice. Similarly, there were enhanced levels of CCL2 and CCL5 in inflamed mice of both groups, however there was no significant difference (Figure 6A,B).

**Figure 7:**
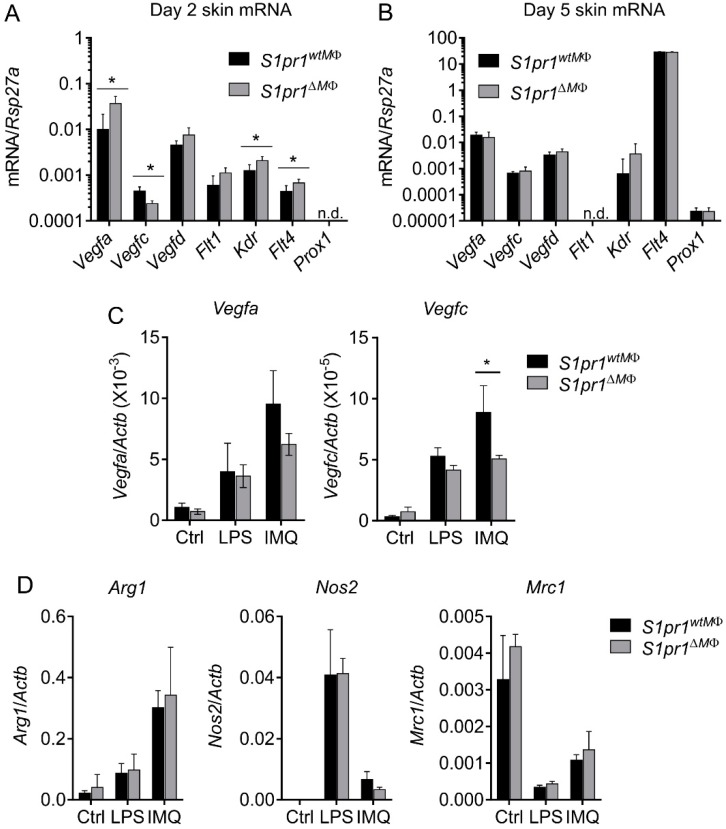
Myeloid S1PR1 is involved in the regulation of VEGFs and their receptors but not in macrophage polarization. (**A**,**B**) *S1pr1^wtMΦ^* (black bar) and *S1pr1^∆MΦ^* (grey bar) mice were treated daily with 62.5 mg IMQ on the back skin for up to 5 days. Gene expression analysis by qPCR for VEGF-A, VEGF-C, VEGF-D, VEGF-R1, VEGF-R2, VEGF-R3 and PROX-1 on whole back skin at day 2 (**A**) and day 5 (**B**). Data are means ± SD, *n* = 4–8 individual animals. Bone marrow-derived macrophages were stimulated with 100 ng/mL lipopolysaccharide (LPS) and 10 μg/mL IMQ for 16 h and mRNA expression of (**C**) VEGF-A and VEGF-C, and (**D**) macrophage polarization markers ARG1, iNOS (*Nos2*) and CD206 (*Mrc1*) was analyzed by qPCR. Data are means ± SD, *n* = 4 individual animals. * *p* < 0.05; *p* values were calculated using two-tailed multiple *t*-test with Holm–Šídák correction (A–C).

Inflammation-driven angiogenesis and lymphangiogenesis can be attributed to the action of inflammatory cytokines secreted from effector cells. Interestingly, IL-1β, which was altered in our mouse model during psoriasis, directly or indirectly influences the expression of various VEGFs and their receptors. The Janus-faced action of IL-1β in angiogenesis [31,32] and lymphangiogenesis [9,12] compelled us to investigate VEGFs and their receptors to understand altered neoangiogenesis and lymphangiogenesis. We measured the relative expression of distinct genes in day two psoriatic skin. In line with the phenotype noted in Figure 4, we observed significantly elevated expression of *Vegfa*, *Kdr* (encoding VEGFR-2), and *Flt4* (encoding VEGFR-3), whereas expression of *Vegfc* was downregulated (Figure 7A). The expression of *Vegfd* and *Flt1* (encoding VEGFR-1) remained unaltered (Figure 7A). However, at this early time point, expression of the master transcription factors of lymph vessels (*Prox1*) was not detectable in whole skin cDNA. Enhanced blood vessel density in psoriatic *S1pr1^∆MΦ^* mice could be explained by the enhanced expression of *Vegfa* along with its receptors *Flt1* and *Kdr*. Conversely, downregulation of *Vegfc*, a lymphangiogenic factor, despite its receptor (*Kdr*; angiogenic and *Flt4*; lymphangiogenic) being expressed, might explain the reduced neolymphatic vessels formation in these mice during skin inflammation. As expected, we did not observe significant differences in these genes in psoriatic ear tissue of these mice (Figure S5E), which may explain why no alterations of ear angiogenesis and lymphangiogenesis were observed (Figure S5A–C). During the resolution phase of inflammation, i.e., day five post IMQ-treatment, the expression of most Vegfs was blunted, except *Flt4*, which was dramatically upregulated, albeit to a similar degree in both strains, whereas the expression of *Flt1* was non-detectable (Figure 7B).

Macrophages also secrete VEGF-A and VEGF-C upon activation. To confirm the non-redundant role of macrophage S1PR1 in skin inflammation, bone marrow-derived macrophages from *S1pr1^∆MΦ^* and *S1pr1^wtMΦ^* were stimulated with LPS and IMQ, to analyze *Vegfa* and *Vegfc* expression. Interestingly, the expression of *Vegfc* was significantly reduced in *S1pr1^∆MΦ^* macrophages upon IMQ-stimulation, whereas *Vegfa* expression remained unaltered in both types of macrophages (Figure 7C). IMQ, which was the stimulus used in the mouse model, was more potent in inducing *Vegf* gene expression than LPS. Furthermore, the expression of *Vegfa* was ~100-fold higher than the expression of *Vegfc*, which supports the critical role of macrophages as a potential source of VEGF-A in an inflammatory skin microenvironment. Lastly, since TLR agonists used in the mouse model and for macrophage polarization has been implicated in the pathogenesis of psoriasis [33], we investigated the effect of myeloid-*S1pr1* deletion on macrophage response to LPS and IMQ. There was no alternation in the expression of *Arg1*, *Nos2* and *Mrc1* in BMDMs of *S1pr1^∆MΦ^* and *S1pr1^wtMΦ^* (Figure 7D), suggesting that macrophage S1PR1 might be redundant in TLR agonist mediated phenotypic changes. Conclusively, these results provide an insight towards macrophage S1PR1 in reciprocal regulation of neoangiogenesis and neolymphangiogenesis by altering VEGFs and their receptors in an autocrine and paracrine fashion.

## 4. Discussion

The role of S1P and its receptor in skin inflammation is very well discussed in the literature, albeit, not in the context of this study [34]. S1P and its receptors are exploited in terms of regulating either T cell infiltration [35] into inflamed skin or attenuating antigen presentation by skin DCs to T cells in the lymph node. In addition, S1P directly affects keratinocyte differentiation, which is beneficial during the recovery phase of psoriasis. Serendipitously, all the above phenomena have a skin macrophage component being integrated, which may be explained, at least in part, through the prism of this study as discussed below. We demonstrated that myeloid, specifically macrophage, S1PR1, has protective effects in IMQ-induced skin inflammation in mice by an underlying, reciprocal regulation of neoangiogenesis and neolymphangiogenesis (Figure 4). Excessive S1P production in an inflammatory local skin milieu, either by keratinocytes or endothelial cells, should indiscriminately trigger S1PRs on resident and recruited cells, such as macrophages. S1P-mediated inhibition of keratinocyte hyperproliferation, a dominant feature of psoriasis, in combination with their differentiation, inadvertently also triggers a reparative amplification cascade by activating S1PR1 on macrophages, provoking neolymphangiogenesis that ultimately results in healing of inflamed skin. In the normal course of IMQ-induced psoriasis there is a spontaneous healing (Figure 2C), which is histologically characterized by neolymphangiogenesis (Figure 4B). However, we observed that myeloid-specific deletion of S1PR1 not only attenuated neolymphangiogenesis, but also strongly induced angiogenesis (Figure 4D), which is reported to act as a pro-inflammatory in skin inflammation [36]. In fact, comparing human clinical data from non-lesion skin with psoriatic lesions, we observed a similar signature pattern in terms of lymphangiogenesis and angiogenesis, such as *LYVE-1* and *CD31* expression in correlation with *S1PR1* (Figure 5). Plasma levels of VEGF are also significantly increased in patients with stable chronic plaque psoriasis [37] and erythrodermic psoriasis [38]. Moreover, overexpression of VEGFR-1 and VEGFR-2 in the dermal microvascular endothelium has been reported in psoriasis [39]. In addition, the *VEGFA* gene is located in close vicinity of *PSORS1* at the psoriasis susceptibility locus on chromosome six at 6p21 [40] and the +405 CC “high VEGF-A-producing genotype” is associated with early onset of psoriasis [37], suggesting that a pro-angiogenic factor may influence disease progression. However, therapeutic induction of lymphangiogenesis for facilitating resolution of inflammation must be approached with caution, as newly formed lymph vessels might also serve as a route for increased drainage of unfiltered pathogens and inflammatory mediators [41].

It has been demonstrated that S1P induces lymphatic endothelial cell (LEC) migration and tube formation in vitro via the S1PR1/G_i_/PLC/Ca^2+^ pathway [42,43]. Secretion of angiopoietin-2 (ANG2) from LECs upon stimulation with S1P is much more pronounced compared to secretion from BECs [44,45]. Given that ANG2 is required for lymphatic development [46,47], S1P may act synergistically with ANG2 during lymphangiogenesis. Furthermore, S1P induces lymphangiogenesis in the Matrigel plug assay [42] and can act in an autocrine manner. Mouse LECs with a specific deletion of *Sphk1* lowered S1P from the lymph but not from the serum, and the development of lymphatic capillaries was morphologically disorganized as cell-cell junctions [48]. These results suggest that LEC-derived S1P is required for normal lymphatic patterning, and our study adds the concept that macrophage S1PR1 is an indispensable part of this process. Importantly, during resolution of inflammation, apoptotic cells, along with a battery of factors, also secrete S1P, which affects the phenotype and effector functions of the local phagocytes via S1PR1 triggered signaling. In a transgenic mouse model of inflammation-driven cancer, we have previously shown that macrophage S1PR1 signaling is crucial for lymphangiogenesis by activating the NLRP3 inflammasome and subsequent activation of IL-1β release. We further demonstrated that S1PR1-dependent IL-1β formation by human macrophages promoted lymphangiogenesis in vitro, and S1P acted as a damage-associated molecular pattern, which is a second trigger required for inflammasome activation [12]. The present study suggests that the role of myeloid S1PR1 in lymphangiogenesis is much more complex than previously thought, since we observed enhanced IL-1β secretion (Figure 6A) and Nlrp3 expression (Figure 2G) in *S1pr1^∆MΦ^* psoriatic skin, whereas lymph vessel density was reduced (Figure 5C). This might also point to unexplored mechanisms, independent of IL-1β, by which myeloid S1PR1 participates in neolymphangiogenesis and regulates discordant neoangiogenesis. 

The involvement of monocytes/macrophages in neolymphangiogenesis is a good example of their exemplary plasticity. They contribute in at least two ways; on one side as a source of VEGF-C after appropriate stimulation, on the other side by transdifferentiation into LECs that integrate into the growing capillaries [49], as demonstrated for tumor-associated macrophages [50] and in kidney transplants during organ rejection [51]. However, recent evidence indicates that DCs are also able to increase [52] or suppress [44] lymphangiogenesis. IMQ-treatment has classically been ascribed to activate dermal DCs and keratinocytes that generate pro-inflammatory cytokines. We have demonstrated that, unlike human macrophages (data not shown), mouse macrophages respond to IMQ in vitro at least with *Vegfa* and *Vegfc* induction (Figure 7C). This highlights the possibility of a direct involvement of skin resident macrophages in disease pathology. Of course, infiltration of macrophages predominately depends on CCL2 and other chemokines produced by keratinocytes and activated DCs in response to inflammatory factors produced by activated immune cells, including resident dermal macrophages [53]. Collectively, our study describes a previously underappreciated role of macrophages in psoriasis.

The role of neutrophil S1PR1 cannot be completely ruled out in our model, because the LysM-Cre deletor strain also affects neutrophils [54]. However, the debate towards a role of neutrophils has not been settled yet. There are conflicting studies arguing for but also against the participation of neutrophils in psoriasis [4,55,56]. We observed late neutrophil infiltration in IMQ-induced psoriasis, which implies a critical role only during the late phase of inflammation, and not in the early phenotype changes observed in *S1pr1^∆MΦ^* mice [20,57].

It is not surprising that in skin inflammation S1PR1 is a critical regulator of angiogenesis, due to the fact that it has been demonstrated to be the first G protein-coupled receptor required for blood vessel formation [58]. Furthermore, pharmacological blockage of S1PR1 by FTY720 or scavenging extracellular S1P by an anti-S1P-neutralizing antibody inhibited tumor-induced angiogenesis [59,60]. Reciprocal regulation of neoangiogenesis and lymphangiogenesis by myeloid S1PR1 in inflamed skin is the most striking observation of this study. Although S1PR1 has never been described in regulating this phenomenon, the reciprocal regulation of neoangiogenesis and neolymphangiogenesis is known in the literature in the context of inflammation. It has been documented that during inflammation, the presence of lymphatic vessels attenuates angiogenesis [61]. Taking this into consideration, it would be plausible to expect S1PR1 as a reciprocal regulator of the neovasculature, especially in light of our recent findings that, at least in tumor context, S1PR1 regulates lymphangiogenesis [9,12]. 

Lymphangiogenesis is also a hallmark of cancer, which enables tumor cells to metastasize at distal sites, leading to poor prognosis. On the other hand, the skin inflammation model of psoriasis presents an inverse situation, where neolymphangiogenesis provokes tissue repair, while excessive neoangiogenesis leads to poor prognosis [12,36]. Therefore, it is important to identify molecular mechanisms that trigger and/or regulate lymphangiogenesis and angiogenesis in skin inflammation so that pharmacological intervention can be achieved in psoriasis. 

The implication of the present study may be of importance in treating psoriasis. Most effective interventions so far target TNFα and its signaling cascade, IL-12 and IL-23, and to some extent S1PRs [13]. However, suboptimal efficacy of the treatment and heterogeneous responses argue for the need of better understanding the role and interaction of these molecules. Macrophages, along with other immigrants of inflamed skin, produce TNFα, IL-12, and IL-23 upon activation, and can produce and respond to local S1P [57,62]. Intracellular S1P has a key role in TNFα signaling by targeting the TNF receptor-associated factor 2 [63]. In addition, high concentrations of S1P enhance the response to a suboptimal dose of TNFα [63]. Furthermore, S1P inhibits proliferation of keratinocytes by elevating intracellular calcium and S1P-stimulated keratinocytes are transformed into corneocytes [64]. This effect is utilized in the treatment of psoriasis with calcitriol or calcipotriol. The antiproliferative and differentiation-promoting effect of active vitamin D3, as well as its analog calcipotriol, are mediated via S1P production [65]. Targeting the S1P/S1PR axis has already been approached in clinical trials using Fingolimod, MT-1303, or Ozanimod for the induction of sustained lymphopenia by trapping T cells in lymphatic organs. However, sustained lymphopenia triggered by S1PR1 antagonism may rather by disadvantageous, since T cells are needed for immunity. Thus, our study adds to the better understanding the role of S1P in skin inflammation via S1PR1 on macrophages, and assigns to them a regulatory function in affecting neoangiogenesis and neolymphangiogenesis. 

## Supplementary Materials

The following are available online at https://www.mdpi.com/2073-4409/8/8/785/s1, Figure S1. S1PR1 is downregulated in human psoriatic patients, Figure S2. S1PR1 deletion fidelity in *S1pr1^∆MΦ^* mice, Figure S3. Inflammatory markers in ear skin of psoriatic *S1pr1^∆MΦ^* mice, Figure S4, Myeloid *S1pr1* deletion has no effect on IMQ-induced ear inflammation, Table S1, List of qPCR primers used, and Table S2. Antibody panel for psoriatic skin characterization by flow cytometry.
