# Supplementary material for: Macrophage S1PR1 Signaling Alters Angiogenesis and Lymphangiogenesis During Skin Inflammation
Source: Cells. 2019 Jul 28;8(8):785. doi: 10.3390/cells8080785 (PMC6721555; doi:10.3390/cells8080785)
Supplement: Supplementary file 1 [file cells-08-00785-s001.pdf]

cells

Macrophage S1PR1 Signaling Alters Angiogenesis and Lymphangiogenesis During Skin Inflammation

Shahzad Nawaz Syed [1], Rebecca Raue [1], Andreas Weigert [1], Andreas von Knethen [1,2] and Bernhard Brüne [1,2,3,4,*]

1 Institute of Biochemistry I, Faculty of Medicine, Goethe-University Frankfurt, 60590 Frankfurt, Germany
2 Project Group Translational Medicine and Pharmacology TMP, Fraunhofer Institute for Molecular Biology and Applied Ecology, 60596 Frankfurt, Germany
3 German Cancer Consortium (DKTK), Partner Site Frankfurt, 60590 Frankfurt, Germany
4 Frankfurt Cancer Institute, Goethe-University Frankfurt, 60596 Frankfurt, Germany
* Correspondence: b.bruene@biochem.uni-frankfurt.de; +49-69-6301-7424

Received: 30 June 2019; Accepted: 27 July 2019; Published: 28 July 2019

Supplementary Material

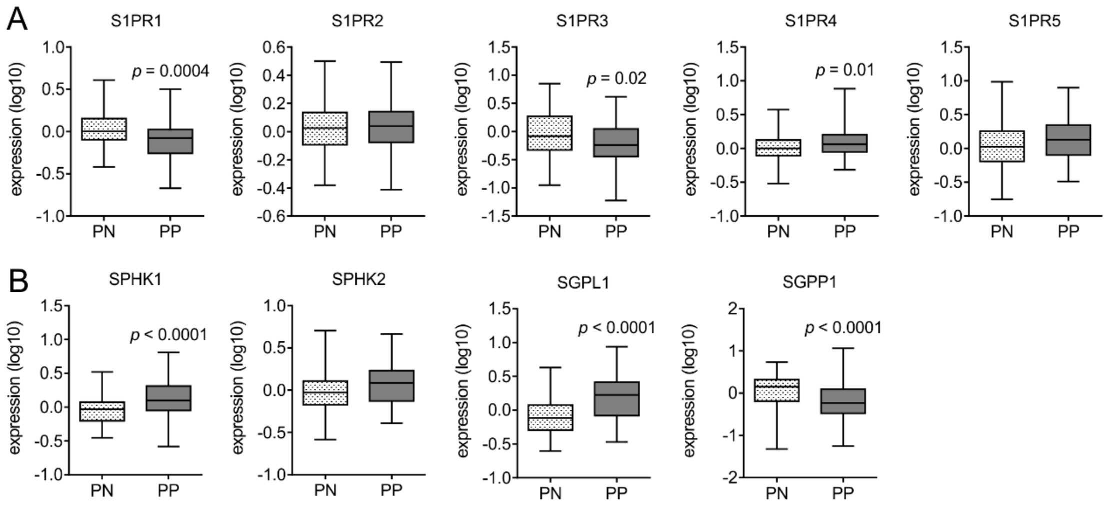

Figure S1: *S1PR1* is downregulated in human psoriatic patients

Gene expression data in Gene Expression Omnibus (GEO) dataset GSE13355 [22] were analyzed for (A) the expression of S1P receptors and (B) S1P metabolizing enzymes in tissues from psoriatic patients with (PP) and without (PN) lesions (n = 58). P values were calculated using two-tailed Student's *t-test*.

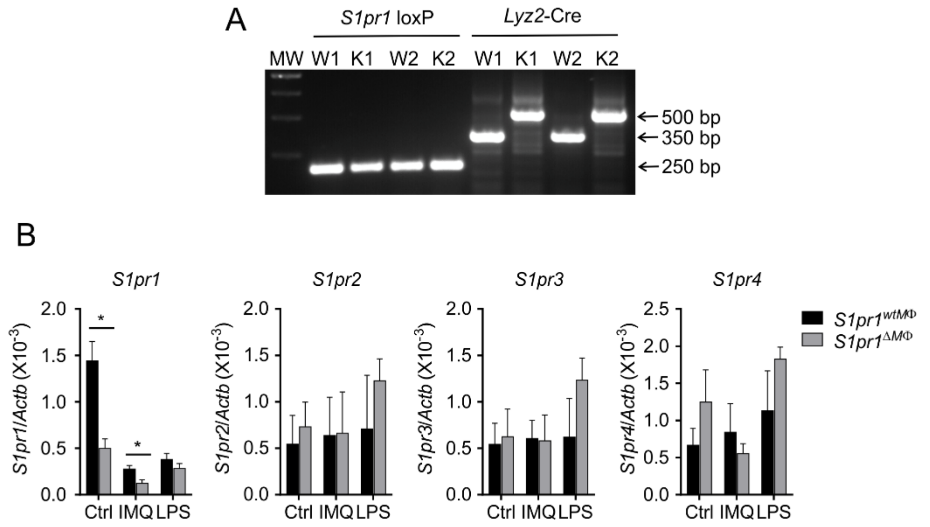

Figure S2: S1PR1 deletion fidelity in *S1pr1*$^{\Delta M\Phi}$ mice

(A) Genotyping PCR was performed on BMDMs from *S1pr1*$^{wtM\Phi}$ (W1 and W2) and *S1pr1*$^{\Delta M\Phi}$ (K1 and K2) mice for loxP site and Cre recombination. Homozygous *S1pr1* floxed cells have a band at ~200 bp, homozygous Cre at ~500 bp, and WT Cre band at ~350 bp. MW = 100 bp ladder. (B) Naïve peritoneal macrophages from *S1pr1*$^{wtM\Phi}$ (black bar) and *S1pr1*$^{\Delta M\Phi}$ (grey bar) mice were treated with 100 ng/mL LPS and 10 µg/mL IMQ for 6 h. mRNA expression of S1P receptors was analyzed by qPCR. Data are means ± SD, *n* = 3-4 individual animals.

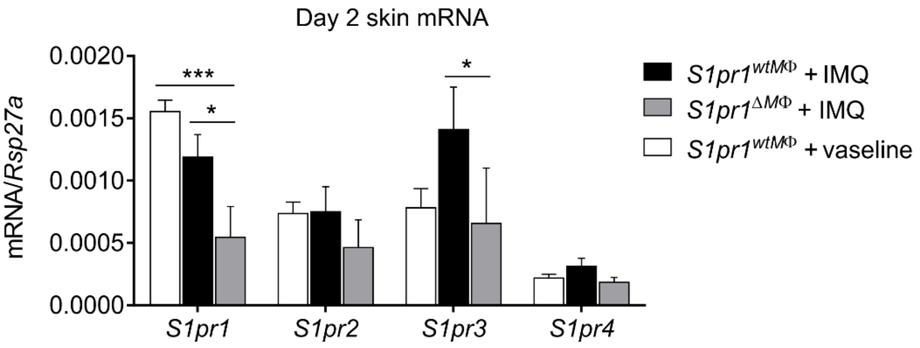

Figure S3: S1P receptors expression in psoriatic back skin of *S1pr1$^{\Delta M\Phi}$* mice

S1pr1$^{wtM\Phi}$ (black bar) and *S1pr1$^{\Delta M\Phi}$* (grey bar) mice were treated daily with 62.5 mg IMQ, compared to controls (S1pr1$^{wtM\Phi}$ + vaseline; white bar) on the back skin for 2 days. mRNA expression of S1PR1-4 was quantified by qPCR in the ear skin. Data are means ± SD, $n = 4$ individual animals. *$p < 0.05$, ***$p < 0.001$; p values were calculated using two-way ANOVA with Turkey's multiple comparison test.

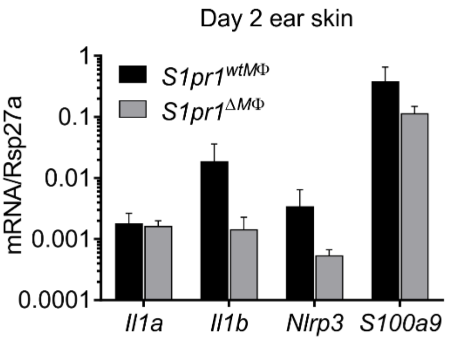

Figure S4: Inflammatory markers in ear skin of psoriatic *S1pr1*$^{\Delta M\Phi}$ mice

S1pr1$^{wtM\Phi}$ (black bar) and *S1pr1*$^{\Delta M\Phi}$ (grey bar) mice were treated daily with 62.5 mg IMQ on the back skin for 2 days. mRNA expression of IL-1α, IL-1β, NLRP3 and S100A9 was quantified by qPCR in the ear skin. Data are means ± SEM, *n* = 4 individual animals.

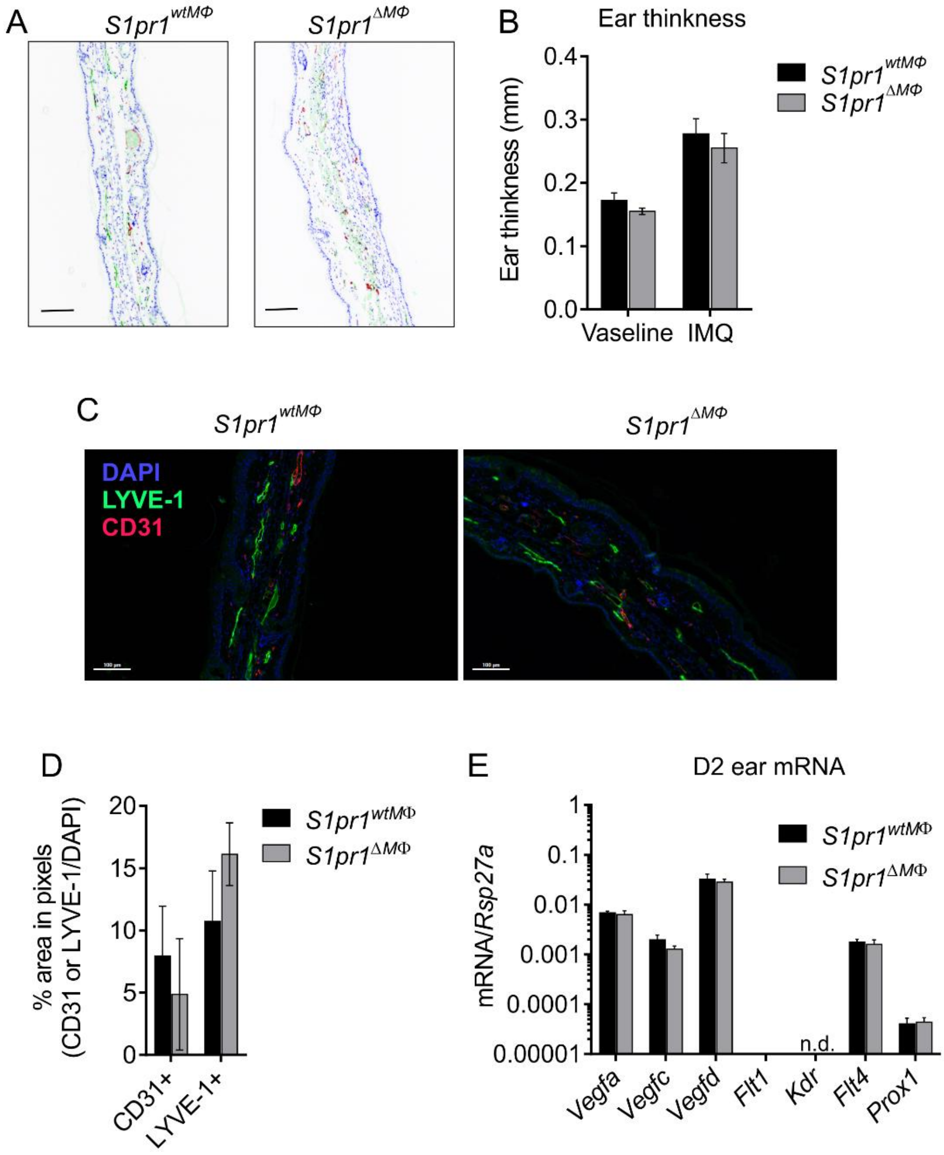

Figure S5: Myeloid *S1pr1* deletion has no effect on IMQ-induced ear inflammation

(A-E) *S1pr1*$^{wtM\Phi}$ (black bar) and *S1pr1*$^{\Delta M\Phi}$ (grey bar) mice were treated daily with 62.5 mg IMQ on the back skin for up to 5 days. (A) Histology images of ear skin sections from 5-day IMQ-treated mice (indicative of 7-9 animals each) stained with anti-LYVE-1 (green), anti-CD31 (red) and DAPI (blue). Scale bars represent 100 µm. (B) Ear thickness at day 5 was measured by caliper of vaseline control or IMQ-treated *S1pr1*$^{wtM\Phi}$ (black bar) and *S1pr1*$^{\Delta M\Phi}$ (grey bar) mice. Data are means ± SEM, n = 4-5 individual animals. (C) PhenOptics images of ear skin sections from 5-day IMQ-treated mice (indicative of 4 animals each) stained with anti-LYVE-1 (green), anti-CD31 (red) and DAPI (blue). Scale bars represent 100 µm. (D) The graph shows quantification of the mean CD31 and LYVE-1 signals in whole ear skin presented as % area in red pixels normalized to blue pixels of nuclear counterstain DAPI. Data are means ± SEM, n = 4 individual animals. (E) Gene expression analysis was performed by qPCR for VEGF-A, VEGF-C, VEGF-D, VEGF-R1, VEGF-R2, VEGF-R3 and PROX-1 on the whole ear skin at day 2.

Table S1: List of qPCR primers used

Gene	forward 5′ - 3′	reverse 5′ - 3′
Il1a	GGGAAGATTCTGAAGAAGAG	GAGTAACAGGATATTTAGAGTCG
Il1b	TGAAATGCCACCTTTTGACA	AGCTTCTCCACAGCCACAAT
Nlrp3	ATTGCTGTGTGTGGGACTGA	AACCAATGCGAGATCCTGAC
S100a9	TCAGACAAATGGTGGAAGCA	GCTCAGCTGATTGTCCTGGT
Vegfd	CCTGGGACAGAAGACCACTC	TGAGATCTCCCGGACATGGT
Flt1	CCTCACTGCCACTCTCATTGTA	ACAGTTTCAGGTCCTCTCCTT
Flt4	GCTGTTGGTTGGAGAGAAGC	TGCTGGAGAGTTCTGTGTGG
Prox1	CGCAGAAGGACTCTCTTTGTC	GATTGGGTGATAGCCCTTCAT
S1pr1	TTGAGCGAGGCTGCTGTTTC	GGGGTGGTATTTCTCCAGGC
Arg1	CTCCAAGCCAAAGTCCTTAGAG	AGGAGCTGTCATTAGGGACATC
Nos2	GTTCTCAGCCCAACAATACAAGA	GTGGACGGGTCGATGTCAC
Actb	CCCTCTGAACCCTAAGGCCA	GGGACAACACAGCCTGGATG
Rsp27a	GACCCTTACGGGGAAAACCAT	AGACAAAGTCCGGCCATCTTC

Table S2: Antibody panel for psoriatic skin characterization by flow cytometry

Marker	Dye	concentration
CD16/32	none	1:50
CD3	PE-CF594	1:100
CD4	BV711	1:100
CD8	BV650	1:200
CD11b	BV605	1:200
CD11c	BV711	1:100
CD19	APC-H7	1:100
CD31	PE-Cy7	1:1000
CD34	FITC	1:100
CD44	AlexaFluor700	1:100
CD45	VioBlue	1:50
CD49f	PE-CF594	1:1000
CD90.2	PE	1:100
CD117	APC-eFluor780	1:200
CD140	PE	1:50
CD146	AlexaFluor488	1:100
CD324	AlexaFluor 647	1:100
CD326	BV711	1:200
GITR	FITC	1:100
SiglecH	FITC	1:100
F4/80	PE-Cy7	1:100
γδ TCR	APC	1:100
HLA-DR (MHC II)	APC	1:200
Ly-6C	PerCP-Cy5.5	1:100
Ly-6G	APC-Cy7	1:100
NK1.1	BV510	1:100

© 2019 by the authors. Submitted for possible open access publication under the terms and conditions of the Creative Commons Attribution (CC BY) license (http://creativecommons.org/licenses/by/4.0/).
